# Inhibitory Activity of Glycosides from *Elsholtzia ciliata* against Soluble Epoxide Hydrolase and Cytokines in RAW264.7 Cells

**DOI:** 10.4014/jmb.2410.10011

**Published:** 2024-11-11

**Authors:** Jang Hoon Kim, Ji Hyeon Park, Kyung-Sook Han, Eun-Song Lee, Yong-Goo Kim, Yong-Il Kim, Sung Cheol Koo, Byoung Ok Cho

**Affiliations:** 1 Department of Herbal Crop Research, National Institute of Horticultural & Herbal Science, RDA, Eumsung, Chungbuk 27709, Republic of Korea; 2 Institute of Health Science, Jeonju University, Jeonju-si 55069, Republic of Korea

**Keywords:** *Elsholtzia ciliata*, Lamiaceae, soluble epoxide hydrolase, glycoside, cytokines

## Abstract

Soluble epoxide hydrolase (sEH) and pro-inflammatory cytokines are associated with the development of inhibitors for cardiovascular and inflammatory diseases. Here, we report on four natural sEH inhibitors isolated from the aerial parts of *Elsholtzia ciliata* (Thunb.) Hyl.. The four compounds, 1–4, were identified as luteolin-7-*O*-glucoside (1), yuanhuanin (2), apigenin-7-*O*-glucoside (3), and butein-4'-*O*-glucoside (4). Among them, compounds 2 and 4 are reported for the first time from this plant. *In vitro* and *in silico*, they showed inhibitory activity towards sEH at micromole concentrations. Moreover, they suppressed pro-inflammatory cytokines in polyinosinic:polycytidylic acid (poly(I:C))-stimulated RAW264.7 cells. Notably, 4 significantly downregulated the sEH catalytic reaction, NO and PGE2 production, and the expression levels of iNOS, COX-2, IL-6 mRNA, and sEH mRNA. Therefore, butein-4'-*O*-glucoside (4) is a potential sEH inhibitor that may be suitable for treating inflammation and cardiovascular diseases caused by infection.

## Introduction

Cardiovascular disease is a leading cause of death worldwide. In 2019, approximately 17 million people died from such disease, accounting for 32% of all global deaths. Overall, 85% of such deaths are due to heart attack and stroke [1]. Arachidonic acid is converted into epoxyeicosatrienoic acids (EETs) by cytochrome P450 epoxygenases [2]. EETs exist as four regioisomers (5,6-, 8,9-, 11,12-, and 14,15-EETs) and are endothelium-derived hyperpolarizing factors [3]. These EETs have anti-apoptotic, anti-inflammatory, vasodilatory, and anti-angiogenic properties [2], inhibiting inflammation by interfering with the transcription of nuclear factor κB into the nucleus [3,4]. Soluble epoxide hydrolase (sEH; EC: 3.3.2.10), a 140 kDa homodimer, is an α/β hydrolase composed of C-terminal and *N*-terminal hydrolases [5]. The former is an enzyme that converts dihydroxyeicosatrienoic acids (DHETs) into a three-member oxirane ring of EETs [5]. The latter phosphorylates isoprenoid phosphates and lysophosphatidic acid, which are involved in cell growth [5]; however, the roles of phosphatase in biological activity are not yet clear [6]. sEH is encoded by the *Ephx2* gene and is widely expressed in tissues including the liver, lungs, kidneys, heart, brain, intestines, and vascular endothelium [7]. Studies on sEH gene knockout mouse models have reported decreases in systolic blood pressure and DHET levels, as well as increases in EETs and survival rates [8]. sEH is composed of Tyr381 and Tyr465, which interact with the oxygen atom of the epoxide and nucleophilic residues (the catalytic triad Asp333-Asp495-His523) in the catalytic pocket [9]. The earliest sEH inhibitors were from chalcone oxides and phenylglycidols [9]. In 1999, the urea skeleton inhibitors *N,N*’-dicyclohexyl-urea (DCU) and *N*-cyclohexyl-*N*’-(3-phenylpropyl)urea were developed [9]. DCU was subsequently improved by adding a flexible side chain, to create 12-(3-adamantan-1-yl-ureido)dodecanoic acid (AUDA) [10]. EC5026 is a urea-type sEH inhibitor developed by EicOsis Human Health Inc. as an immediate-release oral dosage form in a gelatin capsule aimed at treating neuropathic pain [11, 12]. However, such synthetic sEH inhibitor has low solubility in water and are associated with side effects such as aberrant angiogenesis [12, 13]; thus, it is important to identify and study natural sEH inhibitors.

*Elsholtzia ciliata* (Thunb.) Hyl. is an annual plant in the Lamiaceae family found in Korea, China, and Europe [14]. It contains volatile and phenolic compounds and has been used as a medicine and food ingredient [15]. Its constituents include chalcone, phenylpropanoid, flavonoid, and benzoic acid derivatives, which have neuroprotective effects [16]; extracts from this plant have been studied for their potential use to treat cardiovascular diseases, neurodegenerative disorders, obesity, and cancer [15]. In this study, we isolated four natural sEH inhibitors from *E. ciliate*, compounds **1**–**4**. We also characterized their chemical conformations and tested their abilities to inhibit enzyme activity and cytokines levels.

## Materials and Methods

### General Experimental Procedures

One-dimensional dimeter signals were acquired using a AVANCE III-400 spectrometer (Bruker, USA). Analytical thin-layer chromatography (TLC) was performed on precoated normal phase and reversed-phase (Merck, USA) plates, respectively. Column chromatographies were performed using silica gels (60 Å, 230–400 mesh ASTM; Merck, Germany) and C-18 silica gels (ODS-A, S-75 μm; YMC, Japan). The spots developed on the plates were visualized under UV light (254 nm) and 10% H_2_SO_4_, and then heated at 300°C for 30 s. Methanol-*d*_4_ (441384), dimethyl sulfoxide-*d*_6_ (151874), 2-amino-2-(hydroxymethyl)-1,3-propanediol (tris, catalog no. T6066), albumin bovine serum (BSA, A7906), griess reagent, protease inhibitors, and polyinosinic–polycytidylic acid sodium salt (poly(I:C)) were obtained from Merck and human recombinant sEH (10011669), 3-phenyl-cyano(6-methoxy-2-naphthalenyl)methyl ester-2-oxiraneacetic acid (PHOME, 10009134), and AUDA (10007972) were achieved from Cayman Chemical (USA). Fetal bovine serum (FBS) and Dulbecco’s modified Eagle medium (DMEM) were gained from Gibco (USA). Penicillin-streptomycin, trypsin-EDTA (0.25%) PowerTrack SYBR Green Master Mix, and RIPA Lysis and Extraction Buffer were obtained from Thermo Fisher Scientific (USA). EZ-Cytox reagent and EZ-western Kit were purchased from DoGenBio (Republic of Korea). The Bio-Rad Protein Assay was obtained from Bio-Rad (USA). Cyclooxygenase-2 (COX-2) and inducible nitric oxide (iNOS) primary antibodies were sourced from Cell Signaling Technology (USA). β-actin primary antibody and HRP conjugated secondary antibody were procured from Santa Cruz Biotechnology (USA). PGE2 and IL-6 ELISA kits were purchased from R&D Systems (USA). GeneAll Ribospin II extraction kit was obtained from GeneAll Biotechnology (Republic of Korea). The PrimeScript II 1st Strand cDNA Synthesis Kit was sourced from Takara Bio Inc. (Japan) [17].

### Plant Material

The aerial parts of *E. ciliata* were obtained from a herbal remedies shop in Gyeonggi province, Republic of Korea, in March 2023 and were authenticated by Dr. J.H. Kim. A voucher specimen (EC2303–1) was deposited at the herbarium of the Department of Herbal Crop Research, National Institute of Horticultural and Herbal Science, Republic of Korea [17].

### Extraction and Isolation

The *E. ciliata* samples (5 kg) were extracted twice with 36 L of ethanol for a total of 14 days. The extract, condensed under vacuum, was suspended in water and sequentially partitioned into *n*-hexane, ethyl acetate, and water layers. The ethyl acetate fraction was subjected to silica gel column chromatography with a gradient solvent system (CHCl_3_:MeOH, 20:1 to 1:1) to yield fractions E1–E10. Fraction E7 was chromatographed by C-18 column chromatography through a gradient solvent process (H_2_O:MeOH, 2:1 to 1:2) to obtain compound **1** (200 mg) and fractions E71–E75. Compound **2** (98 mg) was isolated from fraction E73 via C-18 column chromatography using an isocratic solvent system (60% MeOH). Fraction E9 was separated via C-18 column chromatography using an isocratic solvent system (55% MeOH) to yield compounds **3** (45 mg) and **4** (29 mg) [15].

### Inhibition of sEH

In 96-well plates, 130 μl recombinant human sEH (100 ng/ml) in 25 mM tris–HCl (pH 7.2) containing 0.1%BSA buffer was added to 20 μl isolated compounds in methanol. Then 50 μl 20 μM substrate (3-phenyl-cyano(6-methoxy-2-naphthalenyl)methyl ester-2-oxiraneacetic acid, PHOME) was added to the mixture. The reactions were monitored for 40 min at 37°C. The product relative fluorescence units (RFU) were measured using a fluorescence photometer (emission wavelength: 465 nm; excitation wavelength: 330 nm). The percentage inhibition was calculated as follows:

Inhibition rate (%) = [(ΔC-ΔI) /ΔC] × 100

where ΔC and ΔI are the differences in the RFU signals of methanol and inhibitor after 40 min, respectively.

y = [(a×x) /(b+x)] + y_0_

where y_0_ is the minimum value on the y-axis, ‘a’ denotes the difference between the maximum and minimum values, and ‘b’ refers to the x value at 50%.

### Molecular Docking Predictions

The 3D structures of the inhibitors luteolin-7-*O*-glucoside (**1**), yuanhuanin (**2**), apigenin-7-*O*-glucoside (**3**), and butein-4'-*O*-glucoside (**4**) were sketched and optimized using Chem3D Pro (CambridgeSoft, USA). 3ANS pdb file stored in the RCSB protein data bank was downloaded. Substrates other than enzyme in data were removed. Hydrogen was added to that by AutoDockTools (Scripps Research, USA) followed by the addition of a Gasteiger charge. For molecular docking, a torsion tree was assigned by detecting torsion roots and rotatable bonds of ligand. For blind docking, the grid box was set to a size containing the entire enzyme (126 × 126 × 126 at 0.375 Å). As a condition for docking, the maximum number of evaluations was used and the Lamarckian genetic algorithm was given. LigPlot (European Bioinformatics Institute, UK) was used to visualize the results [17, 18].

### Molecular Dynamics of Predicted Docking

The Gromacs software was used to simulate the docking result of sEH with butein-4'-*O*-glucoside. A CHARMM all-atom force field was assigned to sEH. Butein-4'-*O*-glucoside was created as an STR file on the GGenFF server and then converted to .gro and .itp files using CHARMM36-ff. The sEH–butane-4'-*O*-glucoside complex was placed inside a cubic box containing H_2_O and sodium using a simple point filling. The simulation was conducted similarly to the previously reported method [18]. Finally, a molecular dynamics was calculated for 100 ns. The result was documented by SigmaPlot (USA) and Chimera (USA) [17, 18].

### Cell Cultures

RAW264.7 cell line was obtained from ATCC (USA). The cells were cultured and maintained in DMEM solution supplemented with 10% FB, and 1% antibiotics (100 U/ml penicillin and 100 μg/ml streptomycin) in a 5%CO_2_ incubator at 37°C.

### Cell Viability

Cell viability was analyzed using EZ-Cytox reagent. The RAW264.7 cells (2 × 10^5^ cells/ml) were seeded into 96-well plates and cultured for 24 h at 37°C. Then the cells were treated with inhibitors (0–50 μM) and further cultured at 37°C. After 24 h incubation, 10 μl EZ-Cytox reagent was added to each well. Subsequently, the absorbance in each well was measured at 450 nm using a spectrophotometer (Tecan Group, Switzerland) after 4 h. The absorbance was correlated with the number of viable cells.

### Inhibitory Activity of Inhibitors on Nitric Oxide Production

RAW264.7 cells (2 × 10^5^ cells/ml) were seeded in 48-well plates for 24 h and pretreated with inhibitors at concentrations of 50 μM for 1 h. Then the cells were stimulated by treating them with 50 μg/ml poly(I:C). After further incubation for 24 h, 100 μl cell supernatant and 100 μl Griess reagent were mixed into 96-well plates. The mixture was incubated for 10 min at room temperature, and the absorbance was determined at 540 nm to quantify NO production. The concentration of NO was determined from a sodium nitrite standard curve developed during the experiment.

### Inhibitory Activity of Inhibitors on PGE2 and IL-6 Production

RAW264.7 cells (2 × 10^5^ cells/ml) were seeded in 48-well plates for 24 h and pretreated with inhibitors at concentrations of 50 μM for 1 h. Then the cells were stimulated by treating them with 50 μg/ml poly(I:C) and further cultured for 24 h. The cell culture supernatant was collected, and then PGE2 and IL-6 production were measured using ELISA assay kits according to the manufacturer’s protocols.

### Inhibitory Activity against iNOS and COX-2 Protein

RAW264.7 cells (2 × 10^5^ cells/ml) were cultured in 6-well cell culture plates for 24 h and pretreated with 50 μM inhibitor. After 1 h, the cells were stimulated with 50 μg/ml poly(I:C) for 24 h. The cells were harvested and lysed using RIPA buffer containing protease inhibitors. After quantification of protein concentrations, 30 μg protein was separated on SDS-polyacrylamide gels at 120 V for 1 h. Then the proteins were transferred onto PVDF membranes at 100 V for 1 h. Subsequently, the membranes were blocked with 5% skim milk, washed with TBS-T buffer, and incubated with COX-2 and iNOS antibodies overnight at 4°C. After washing with TBS-T buffer five times for 5 min each, the membranes were incubated with HRP-conjugated secondary antibodies for 2 h at room temperature. Then they were washed again with TBS-T buffer five times for 5 min and detected with an imaging system (Alliance version 15.11; UVITEC) using an EZ-western Kit. Band densities were analyzed using ImageJ gel analysis software (National Institutes of Health, USA).

### Inhibitory Activity against IL-6 and sEH mRNA Expression

RAW264.7 cells (4 × 10^5^ cells/ml) were cultured in 60 mm cell culture dishes for 24 h in a humidified incubator at 37°C, 5% CO_2_. The cells were pretreated with 50 μM inhibitor for 1 h, followed by continued culture for an additional 3 h with poly(I:C) (50 μg/ml) stimulation. Total RNA was extracted using the GeneAll Ribospin II extraction kit and reverse transcription was performed using the PrimeScript II 1st Strand cDNA Synthesis Kit in a T100 Bio-Rad Thermal Cycler. A StepOne Real-Time PCR system (Thermo Fisher Scientific) and SYBR were used for real-time PCR amplification for IL-6, sEH, and GAPDH under the following conditions: 95°C for 1 min and 40 cycles of amplification at 95°C for 30 s, 60°C for 30 s, and 72°C for 30 s. The primers used for PCR analysis for IL-6 were 5’-TCCAGTTGCCTTCTTGGGAC-3’ (forward) and 5’-ACAGGTCTGTTGGGAGTGGT-3’ (reverse); those for sEH were 5’-GGACGACGGAGACAAGA- GAG-3’ (forward) and 5’-CTGTGTTGTGGACCAGGATG-3’ (reverse); and those for GAPDH were 5’-GGCTACACTGAGGACCAGGT-3’ (forward) and 5’-TCCACCACC CTGTTGCTGTA-3’ (reverse). Expression levels were normalized to GAPDH using the 2-ΔΔCt method.

### Statistical Analysis

The results are presented as means ± SDs. Statistical comparisons were performed using IBM SPSS Statistics 23 (IBM, USA). Statistically significant differences among groups were determined using one-way analysis of variance (ANOVA) followed by a Tukey’s test. A *p* value < 0.05 was considered statistically significant.[Fig F1]

## Results

### Extract and Isolation of the Aerial Parts of *E. ciliata*

Samples of *E. ciliata* were extracted with ethanol, *n*-hexane, ethyl acetate, and water. The ethyl acetate fraction was purified on a silica gel-based C-18 column, resulting in the isolation of the four compounds luteolin-7-*O*-glucoside (**1**) [19], yuanhuanin (**2**) [20], apigenin-7-*O*-glucoside (**3**) [19], and butein-4'-*O*-glucoside (**4**) [21]. Their structures were elucidated by comparing them to previous reports (Fig. S1–S4).

### Inhibitory Activity and Enzyme Kinetics of sEH

**Fig. 2:**
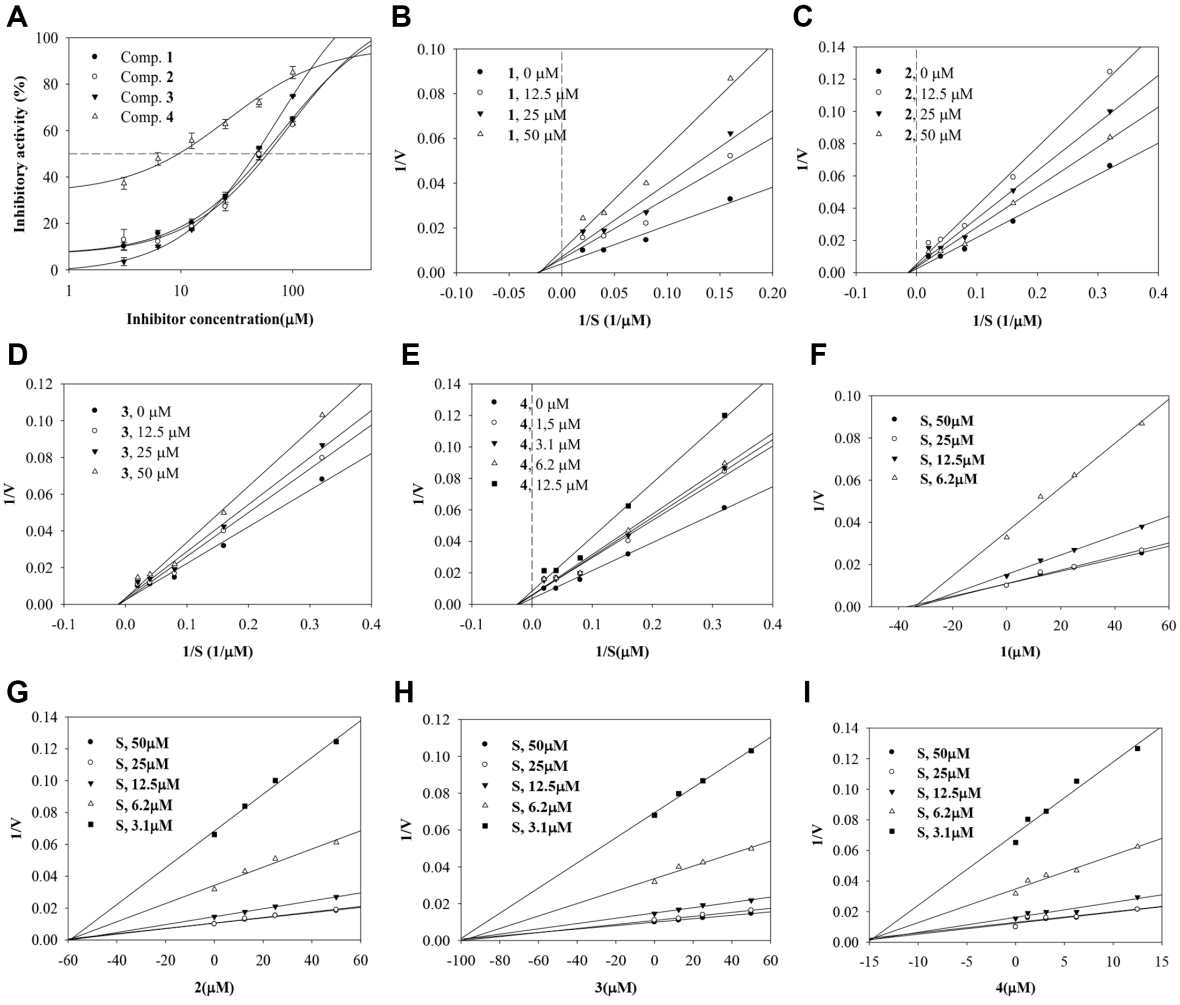
The inhibitory activity (A), Lineweaver-burk (B–E) and Dixon (F–I) plots of compounds 1–4 toward sEH.

Compounds **1**–**4** were evaluated for their ability to block the catalytic reaction of sEH using PHOME. Their inhibitory rates were calculated using equation 1. The ethanol extract showed 100% inhibitory activity. IC_50_ values were calculated using equation 2; they indicated inhibition in a dose-dependent manner. The enzyme kinetics of compounds **1**–**3** were investigated at concentrations ranging from 12.5 to 50 μM, while compound **4** was studied at 1.25–12.5 μM (Fig. 2A). The substrate-enzyme initial velocity (*v*_0_) of the compounds was determined at substrate concentrations ranging from 3.1 to 50 μM. As shown in Fig. 2B-2I and Table 1, the results indicated noncompetitive inhibitors with inhibition constants (*k*_i_) of 98.2, 56.9, 34.1, and 14.3 μM, respectively.

### Molecular Simulation

**Fig. 3:**
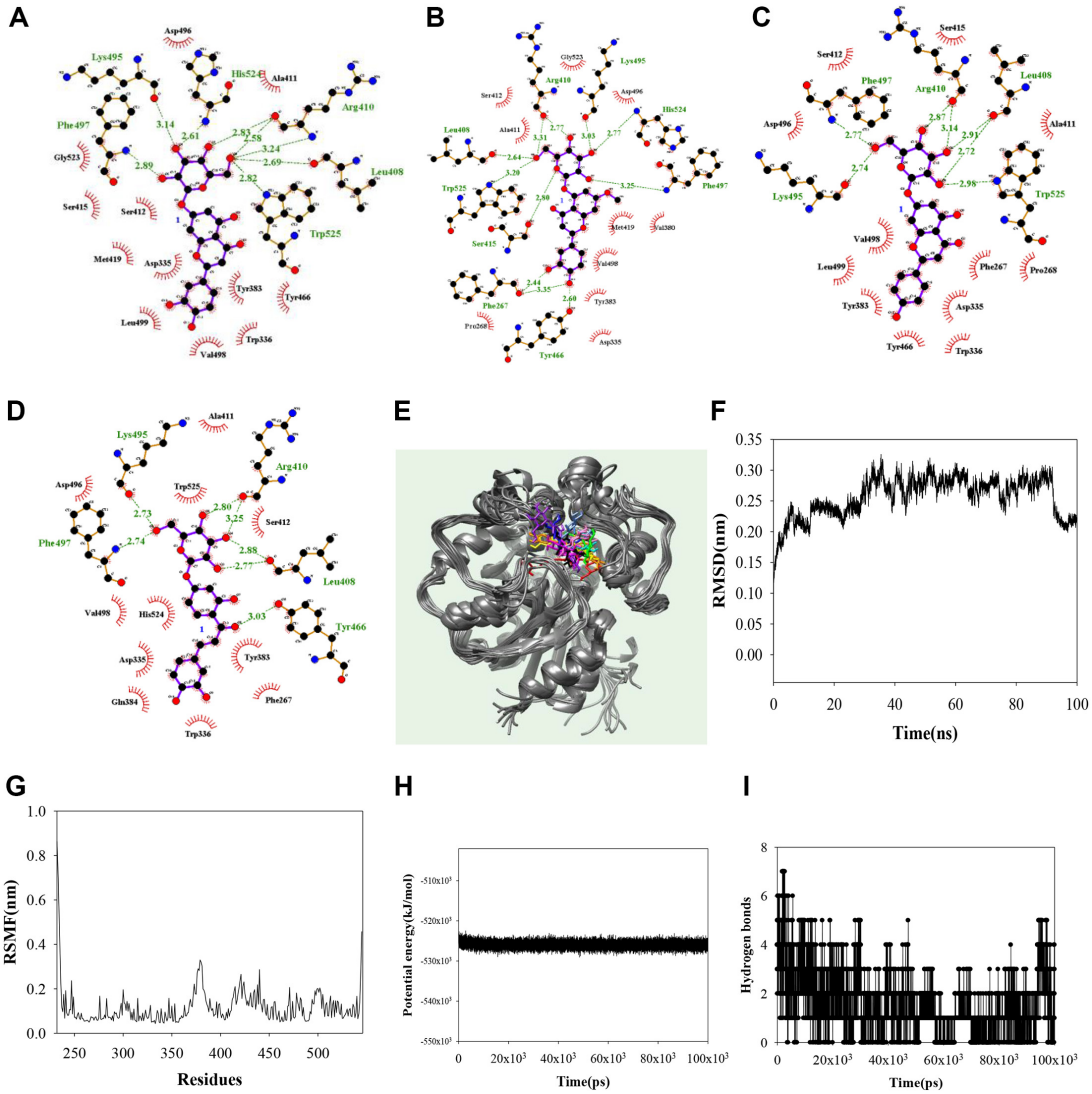
The hydrogen bonds of inhibitors 1–4 (A–D) with sEH. The overlapped pose of inhibitor **4** (**E**) with sEH for 100 ns (red: 0 ns, orange: 10 ns, yellow: 20 ns, green: 30 ns, cyan: 40 ns, blue: 50 ns, conflower blue: 60 ns, purple: 70 ns, menganta: 80 ns, white: 90 ns, black: 100 ns). RMSD (**F**), RMSF (**G**), the potential energy (**H**), and hydrogen bonds (**I**) of the simulation.

Noncompetitive inhibitors were simulated using a blind docking method to identify the binding sites. As shown in Fig. 3A–3D and Table 2, the AutoDock scores were -10.22, -10.00, -9.14, and -9.14 kcal/mol for compounds **1**–**4**, respectively. The hydroxyl group in the glycone of compound **1** formed hydrogen bonds with Leu408 (2.72 and 2.91 Å), Arg410 (2.87 and 3.14 Å), Lys495 (2.74 Å), Phe497 (2.77 Å), and Trp525 (2.98 Å). That of compound 2 did with Phe267 (2.44, 3.35 Å), Leu408 (2.64 Å), Arg410 (2.77, 3.31 Å), Ser415 (2.80 Å), Tyr466 (2.60 Å), Lys495 (3.03 Å), Phe497 (3.25 Å), His524 (2.77 Å), and Trp525 (3.20 Å), and that of compound 3 for did with Leu408 (2.69 Å), Arg410 (2.58, 2.83, and 3.24 Å), Lys495 (3.14 Å), Phe497 (2.89 Å), His524 (2.61 Å), and Trp525 (2.82 Å). The ketone in the aglycone and hydroxyl groups of compound 4 interacted with Tyr466 (3.03 Å), and Leu408 (2.77 and 2.88 Å), Arg410 (2.80 and 3.25 Å), Lys495 (2.73 Å), and Phe497 (2.74 Å). Compound 4 was revealed to be a noncompetitive inhibitor, stably docked with sEH. Next, the **4**–sEH complex was analyzed via molecular dynamics to gain insights into the interactions between the inhibitor and sEH. The results are given in Fig. 3E–3I. The potential inhibitor 4 was visually confirmed to maintain a stable bond between Asp496–Leu499 and Phe409–Met419 loops, and Pro379-Phe381 α-helax. With this inhibitor, sEH had a potential energy of -5.3 × 10^4^ kJ/mol, with a root mean square deviation (RMSD) value within 3.2 Å and a root mean square fluctuation (RMSF) value within 3.3 Å. During 100 ns simulation, **4** and sEH formed mainly 1–3 hydrogen bonds, sometimes 0, or 4–7 hydrogen bonds. At the start of the simulation, it can be confirmed that the inhibitor 4 is bound to the activity site and next to it. It was confirmed that this was stably bound to all cavity next to the active site from 30ns (Fig. 3E).

### Inhibition of NO Production

**Fig. 4:**
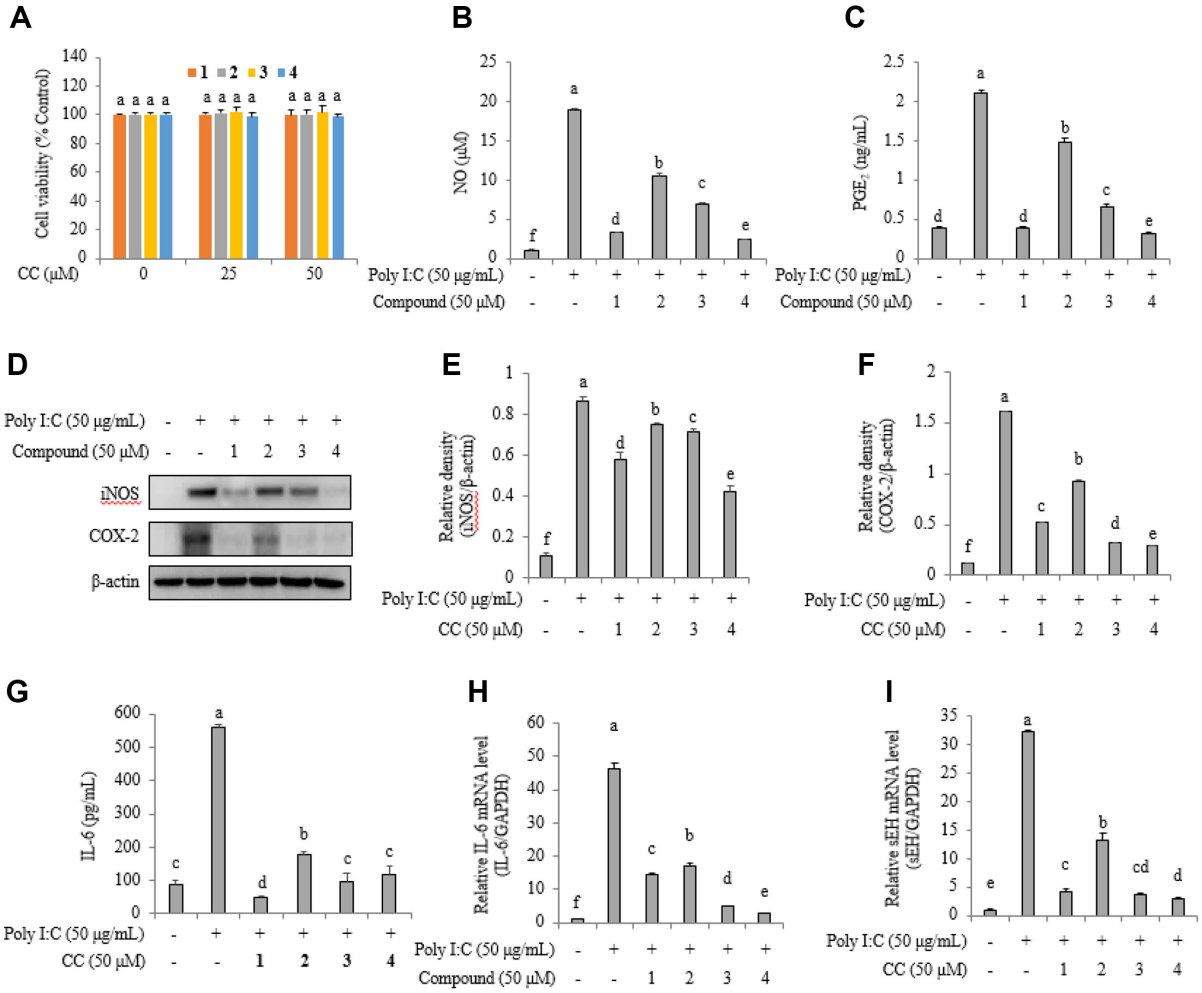
Cell viability of inhibitors on RAW264.7 cells (A). The inhibitory activity of inhibitors **1**–**4** on NO (**B**) and PGE_2_ (**C**) production in poly (I.C.)-stimulated RAW264.7 cells. iNOS and COX-2 protein levels (**D**) in western blot and relative densities iNOS (**E**) and COX-2 (**F**) of by inhibitors. IL-6 (**G**) protein levels, and IL-6 (**H**) and sEH (**I**) mRNA expression levels.

Cytotoxicity tests were performed to determine the concentration at which 1–4 would not affect RAW264.7 cells. They demonstrated cell viability (Fig. 4A). Normal cells secreted 0.93 ± 0.19 μM NO and 0.38 ± 0.01 ng/ml PGE2, whereas poly(I:C)-stimulated cells produced 18.83 ± 0.24 μM NO and 2.10 ± 0.03 ng/ml PGE2. Poly(I:C)-stimulated cells treated with 50 μM. **1**–**4** exhibited NO levels of 3.30 ± 0.04, 10.45 ± 0.39, 6.89 ± 0.24, and 2.36 ± 0.07 μM, and PGE2 levels of 0.38 ± 0.02, 1.48 ± 0.04, 0.65 ± 0.04, and 0.31 ± 0.02 ng/ml, respectively (Fig. 4B and 4C). Western blotting analysis showed a decrease in iNOS and COX-2 protein levels in cell mixtures treated with inhibitors (Fig. 4D). The relative densities of iNOS protein levels in normal cells, poly(I:C)-stimulated cells, and cells treated with **1**–**4**, were 0.10 ± 0.00, 0.86 ± 0.02, 0.58 ± 0.03, 0.75 ± 0.01, 0.71 ± 0.01, and 0.41 ± 0.03, respectively (Fig. 4E). See Figure 4F–4I for more details. Of note, **1**–**4** downregulated COX-2 protein, IL-6 protein, IL-6 mRNA levels, and sEH mRNA levels in poly(I:C)-stimulated cells.

## Discussion

sEH is found in cytosol and peroxisome of liver, kidney, lungs, heart, brain, spleen, adrenals, and intestine tissues [9]. It has been reported that epoxy-fatty acids (EpFAs), which are substrate of sEH, are effective in improving cardiovascular diseases, inflammation, cancer and inflammatory pain [22]. In complete Freund’ adjuvant-induced thermal hyperalgesia rats, TPPU is known to improve inflammatory pain by reducing the concentration of 12,13-dihydroxy-9Z-octadecenoic acid [24]. Among sEH inhibitors, AR9281 and GSK2256294A have being developed up to phase 1 clinical trials to treat hypertension and lung disease, respectively [23]. EpFAs have been unveiled to have the function which suppressed pro-inflammatory cytokines [24]. The Asian plant *E. ciliata* is used as a vegetable, spice, herbal tea, and traditional medicine used to treat colds, fever, and asthma [25]. Its extract exhibits anti-inflammatory activity in RAW264.7 cells and may downregulate interstitial fibrosis [19]; it also increases cell viability in HT22 murine hippocampal cells [16]. Ethanolic extracts from this plant contain polyphenols and have shown anti-inflammatory effects [17]. Extracts commonly contain chlorogenic acid, rutin, luteolin-7-*O*-glucoside, 7-avicularin, 8-apigenin-7-*O*-glucoside, and quercitrin. In the present study, we isolated four glycosides (**1**–**4**) from this plant; they were identified as luteolin-7-*O*-glucoside (**1**), yuanhuanin (**2**), apigenin-7-*O*-glucoside (**3**), and butein-4'-*O*-glucoside (**4**). Among them, compounds **2** and **4** are reported for the first time as components of this plant.

The role of sEH inhibitors in increasing levels of EETs is critical for treating cardiovascular and inflammatory conditions [3]. Research has focused on discovering inhibitors of sEH from synthetic or natural sources [9]. The four compounds exhibited inhibitory activity against sEH as noncompetitive inhibitors. The glycone regions of **1**–**4** interacted with the amino acid residues Leu408, Arg410, Lys495, and Phe497, while the aglycone regions were positioned near the catalytic site of sEH. Notably, **4** maintained a stable binding movement while preserving the glucoside bond with these amino residues during molecular dynamics simulations. Glycoside (**4**) had a better inhibitory effect than the similar aglycone forms, isoliquiritigenin and 2'-methoxyisoliquiritigenin [26]. Chalcone oxides were initially developed as sEH inhibitors [27].

In a previous study on sEH gene deletion mice, pulmonary edema and inflammation caused by hyperoxic acute lung injury were reduced [28]. In addition, levels of nuclear-factor erythroid 2-related factor, heme oxygenase-1, and superoxide dismutase were higher than in normal mice. The sEH inhibitor (*t*-TUCB) reduces inflammatory cell infiltration into the airway and lungs and increases levels of anti-inflammatory mediators such as EETs, dihydroxyoctadecenoic acids, and LTB_4_ [29]. Compounds **1**–**4** demonstrated the ability to decrease the production and expression of anti-inflammatory agents including NO, PGE2, iNOS, COX-2, IL-6, and mRNA levels of IL-6 and sEH in RAW264.7 cells at 50 μM concentration. They suppressed not only the sEH catalytic reaction but also sEH mRNA levels, with 4 having the greatest effect. Poly(I:C), interacts with toll-like receptor 3 and RIG-I-like receptors, promoting the secretion of cytokines, chemokines, and costimulatory factors as if infected with a virus [30].

## Conclusion

Four compounds, **1**–**4**, were purified from *E. ciliate* and were identified as luteolin-7-*O*-glucoside (**1**), yuanhuanin (**2**), apigenin-7-*O*-glucoside (**3**), and butein-4'-*O*-glucoside (**4**). Compounds **2** and **4** were identified for the first time as components of this plant. These compounds exhibit inhibitory activities against sEH as noncompetitive inhibitors and show similar binding to sEH. In addition, they downregulate NO, PGE2, iNOS, COX-2, IL-6, and the mRNA levels of IL-6 and sEH in poly(I:C)-stimulated RAW264.7 cells. Ultimately, compound **4** exhibited the most effective inhibition of sEH and cytokines and was validated as a fully qualified lead compound and potential inhibitor for the development of anti-inflammatory agents related to infection.

## Supplemental Materials



## Figures and Tables

**Fig. 1 F1:**
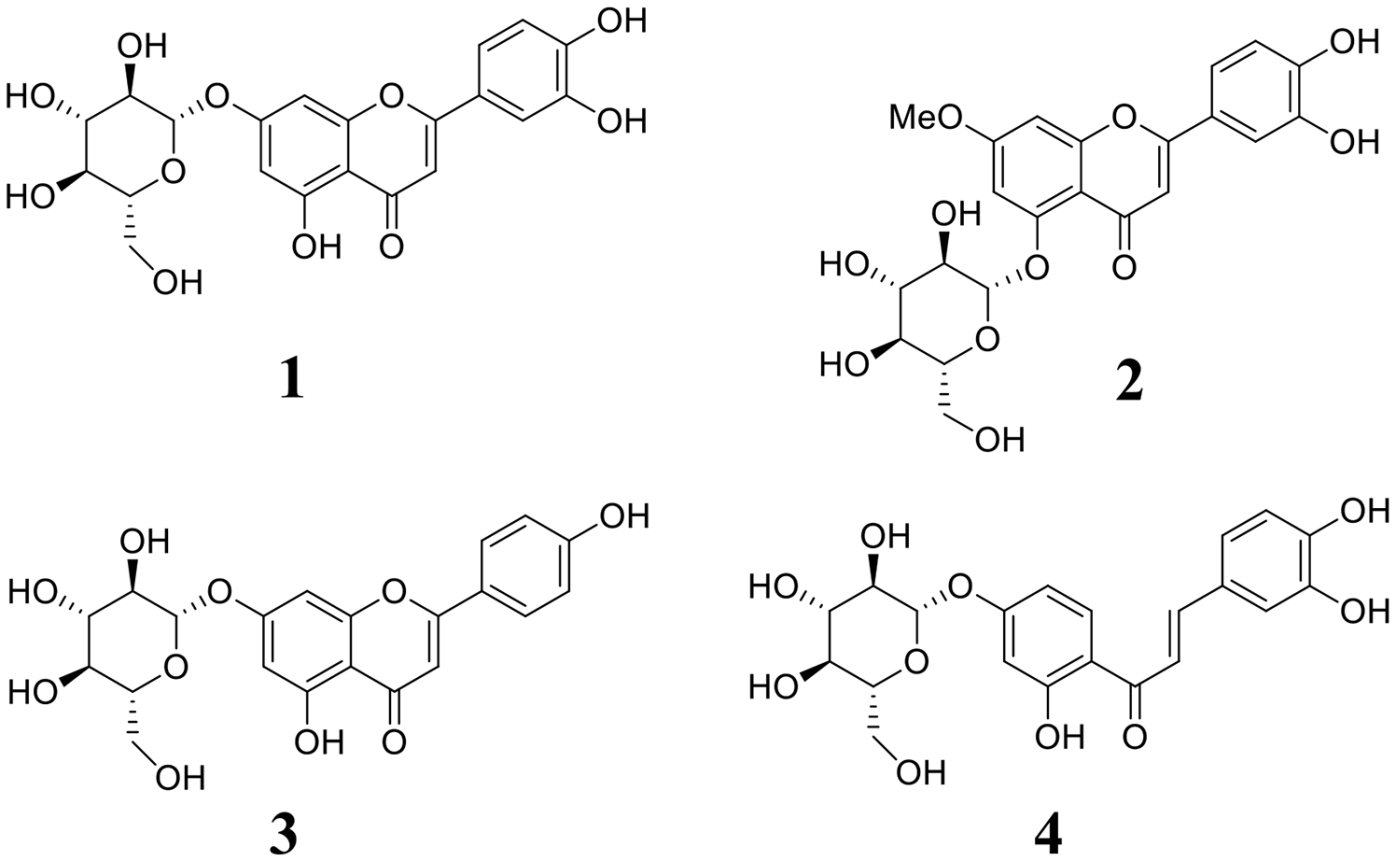
The structure of compounds 1–4 derived from the aerial parts of *E. ciliata*.

**Table 1:** The inhibitory activity of inhibitors from the aerial parts of *E. ciliata* toward sEH.

	100 μM(%)	IC_50_ (μM)	Bindig mode (*k*_i_, μM)
**1**	64.9 ± 0.1	46.7 ± 0.5	Non-competitive (34.1)
**2**	62.5 ± 0.2 6	59.9 ± 0.7	Non-competitive (56.9)
**3**	72.9 ± 1.0	56.3 ± 0.9	Non-competitive (98.2)
**4**	85.0 ± 0.6	9.6 ± 2.1	Non-competitive (14.3)

**Table 2:** Molecular docking of inhibitors derived from the aerial parts of *E. ciliata* with sEH.

	Autodock Score (kcal/mol)	Hydrogen bonds
**1**	-9.14	Leu408(2.69), Arg410(2.58,2.83), His524(2.61), Lys495(3.14), Phe497(2.89), Trp525(2.82)
**2**	-9.84	Leu408(2.64), Trp525(3.20), Ser415(2.80), Phe267(2.44,3.35), Tyr466(2.60), Phe497(3.25), His524(2.77), Lys495(3.03) Arg410(2.77, 3.31)
**3**	-10.23	Leu408(2.72,2.91), Arg410(2.87,3.14), Phe497(2.77), Lys495(2.74), Trp525(2.98)
**4**	-10.22	Leu408(2.77, 2.88), Arg410(2.80, 3.25), Tyr466(3.03), Lys495(2.73), Phe497(2.74)
