# Supplementary material for: Inhibitory Activity of Glycosides from Elsholtzia ciliata against Soluble Epoxide Hydrolase and Cytokines in RAW264.7 Cells
Source: J Microbiol Biotechnol. 2024 Nov 11;35:e2410011. doi: 10.4014/jmb.2410.10011 (PMC11813350; doi:10.4014/jmb.2410.10011)
Supplement: Supplementary file 1 [file jmb-35-e2410011-supple.pdf]

J. Microbiol. Biotechnol. 2025. 35: e2410011

https://doi.org/10.4014/jmb.2410.10011

Supplementary Figures

The Inhibitory Activity of Glycosides from *Elsholtzia ciliata* on Soluble Epoxide Hydrolase and Cytokines in RAW264.7 Cells

Jang Hoon Kim[1], Ji Hyeon Park[2], Kyung-Sook Han[1], Eun-Song Lee[1], Yong-Goo Kim[1], Yong-Il Kim[1], Sung Cheol Koo[1], Byoung Ok Cho[2]*

[1]*Department of Herbal Crop Research, National Institute of Horticultural & Herbal Science, RDA, Eumsung, Chungbuk, 27709, Republic of Korea*

[2]*Institute of Health Science, Jeonju University, 303 Cheonjam-ro, Wansan-gu, Jeonju-si, Jeollabuk-do 55069, Republic of Korea*

* Corresponding Author: Byoung Ok Cho,

enzyme21@jj.ac.kr

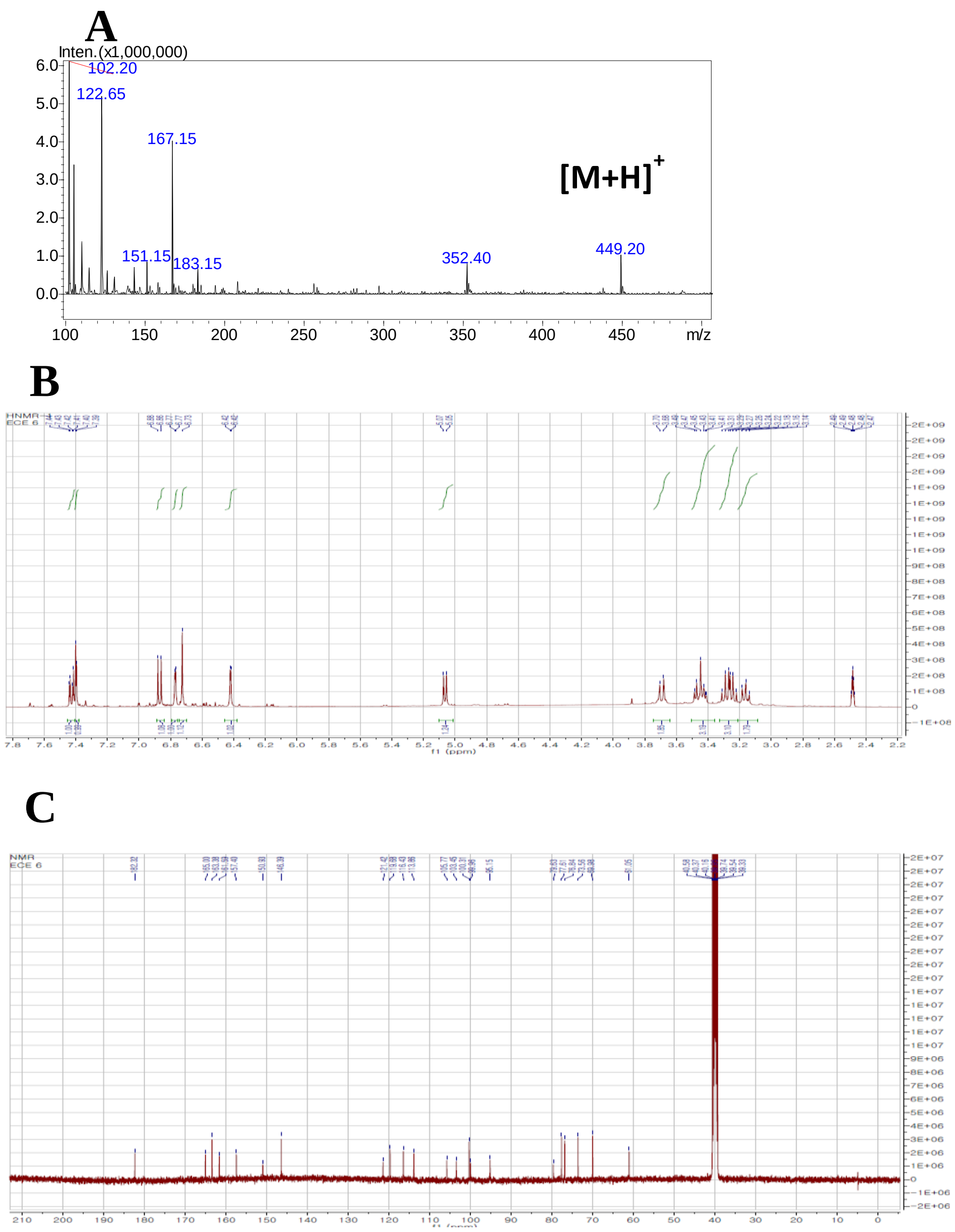

Fig. S1. Mass (**A**) ^{1}H-NMR (**B**) and ^{13}C-NMR (**C**) spectra of compound **1**.

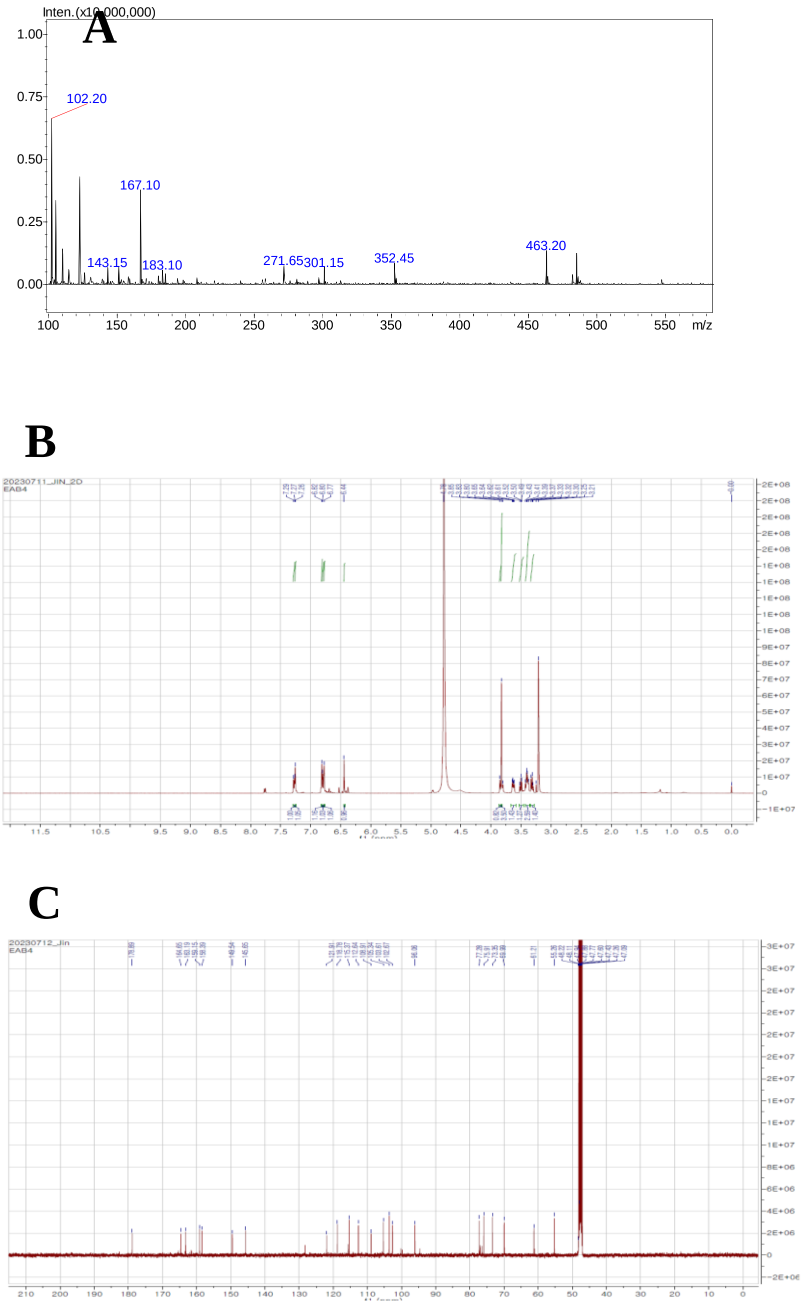

Fig. S2. Mass **(A)** ^{1}H-NMR **(B)** and ^{13}C-NMR (**C)** spectra of compound **2**.

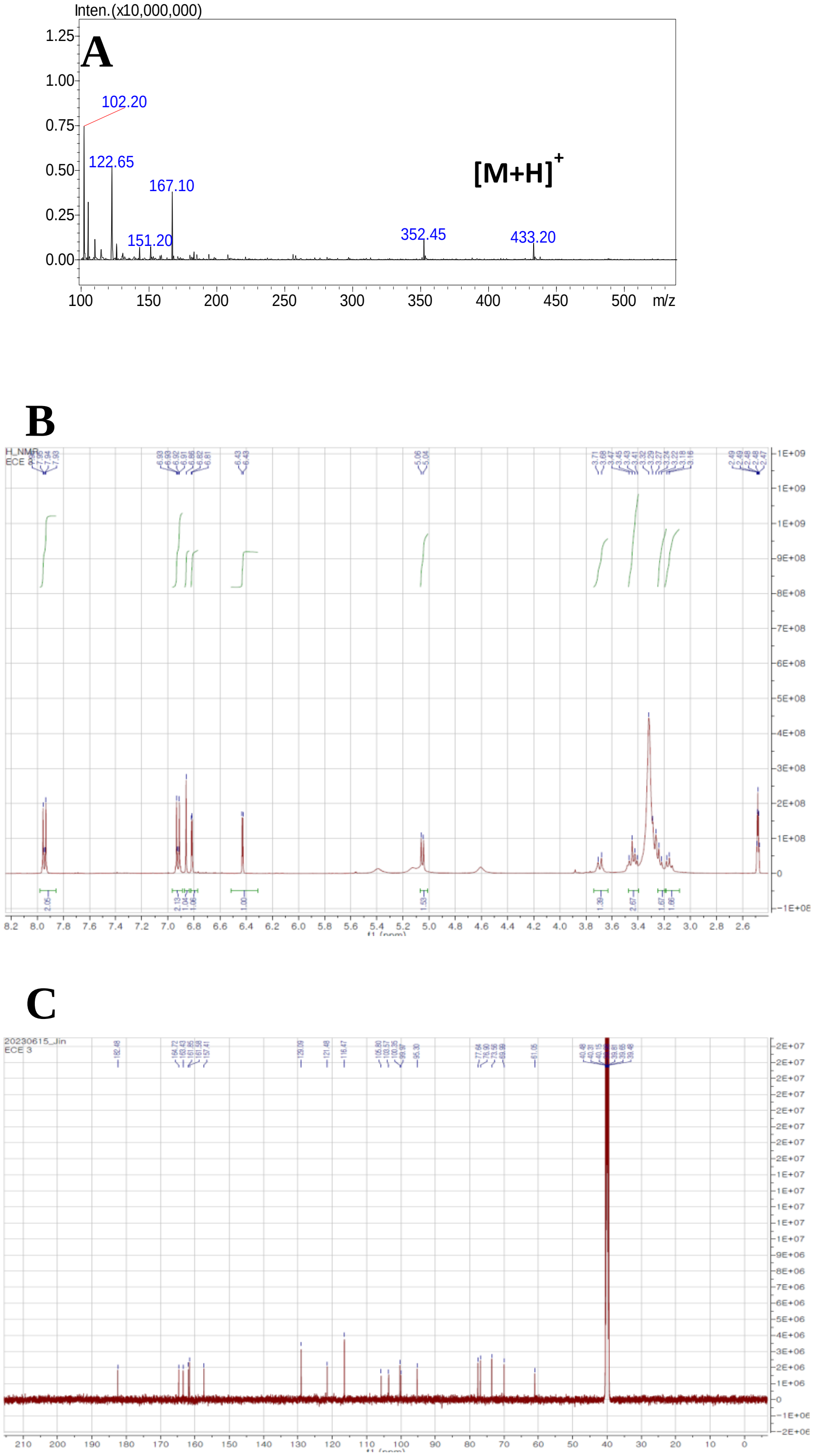

Fig. S3. Mass **(A)** ^{1}H-NMR **(B)** and ^{13}C-NMR (**C)** spectra of compound **3**.

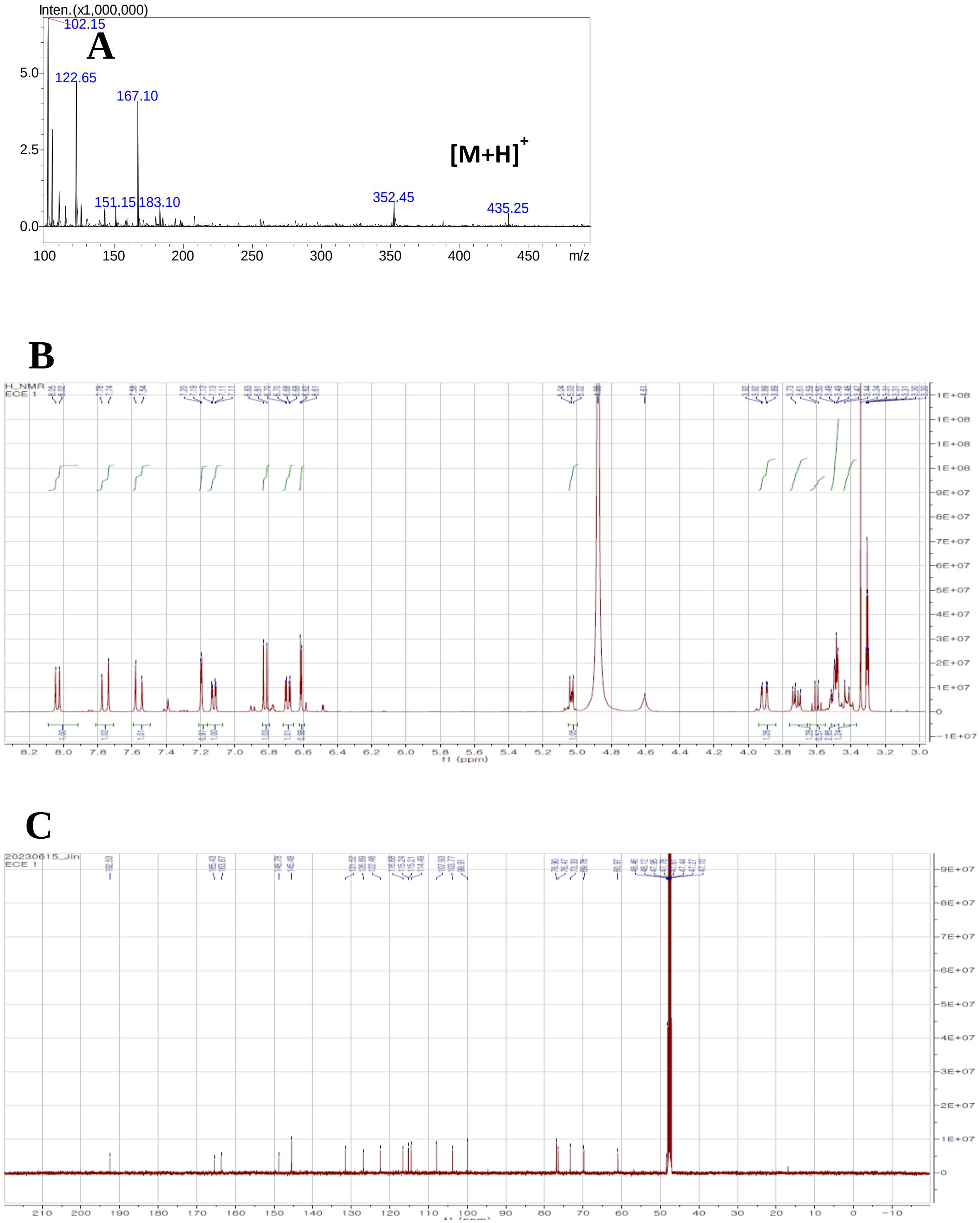

Fig. S4. Mass **(A)** ^{1}H-NMR **(B)** and ^{13}C-NMR (C) spectra of compound **4**.
